# Mechanisms of Hydroxyurea-Mediated DNA Damage Potentiation in Fanconi Anemia Cells

**DOI:** 10.3390/ijms27156782

**Published:** 2026-07-29

**Authors:** Benjamin Bustamante, David Sosa, Jorge Melendez-Zajgla, Gabriel López-Velázquez, Patricia Ostrosky-Wegman, Alfredo Rodriguez, Leda Torres, Ulises Juárez-Figueroa, Roberto Sánchez-Olea, Elsa Cervantes-Ríos, Rocío Ortiz-Muñiz, Bertha Molina, Sara Frias

**Affiliations:** 1 Laboratorio de Citogenética, Instituto Nacional de Pediatría, Insurgentes Sur 3700 C, Col. Insurgentes Cuicuilco, Mexico City 04530, Mexico; benjaminbte@gmail.com (B.B.); david.sosa@lagem.com.mx (D.S.); ledatorres@ciencias.unam.mx (L.T.); ujuarezf@pediatria.gob.mx (U.J.-F.); 2 Doctorado en Biología Experimental, Universidad Autónoma Metropolitana-Iztapalapa, Mexico City 09310, Mexico; 3 Laboratorio de Análisis Genéticos Especializados México (LAGEM), Mexico City 04730, Mexico; 4 Functional Cancer Genomics Laboratory, Instituto Nacional de Medicina Genómica (INMEGEN), Mexico City 14610, Mexico; jmelendez@inmegen.gob.mx; 5 Laboratory of Biomolecules and Infant Health, Instituto Nacional de Pediatría, Mexico City 04530, Mexico; glv_1999@ciencias.unam.mx; 6 Departamento de Medicina Genómica y Toxicología Ambiental, Instituto de Investigaciones Biomédicas, Universidad Nacional Autónoma de México, Mexico City 04510, Mexico; ostrosky@biomedicas.unam.mx (P.O.-W.); alfredo.rodriguez@iibiomedicas.unam.mx (A.R.); 7 Laboratorio de Falla Medular y Carcinogénesis, Instituto Nacional de Pediatría, Mexico City 04530, Mexico; 8 Instituto de Física, Universidad Autónoma de San Luis Potosí, San Luis Potosí 78295, Mexico; rsanchez@ifisica.uaslp.mx; 9 Laboratorio de Biología Celular y Citometría de Flujo, Departamento de Ciencias de la Salud, Universidad Autónoma Metropolitana-Iztapalapa, Mexico City 09310, Mexico; elsacerv@xanum.uam.mx (E.C.-R.); arom@xanum.uam.mx (R.O.-M.)

**Keywords:** Fanconi anemia cells, DNA damage response, checkpoint recovery, mitomycin C, hydroxyurea, ribonucleotide reductase

## Abstract

Fanconi anemia (FA) is a rare disease with a deficient homologous recombination DNA repair pathway and high sensitivity to mitomycin C (MMC). In FA cells, hydroxyurea (HU), when applied in the G2 phase of the cell cycle, exacerbates the chromosomal aberrations (CAs) induced by MMC. Here, we study how exposure to HU in the G2 phase results in an increased CAs frequency in FA cells with or without prior treatment with MMC. We performed chromosome aberration analysis, flow cytometry, and mRNA and protein expression in lymphoblastoid cell lines. We found that HU alone induces post-replicative DNA damage in the form of DNA double-strand breaks (DSBs) in both wild-type and FA cells and a similar increase in the activation of DSBs marker ɣH2AX. HU potentiates the amount of CAs only in MMC-treated FA cells by multiple mechanisms, including (1) hindering the ɣH2AX signal, (2) impairing the translocation of ribonucleotide reductase (RNR) into the cell nucleus, and (3) promoting the activity of WIP1 phosphatase. Concomitantly, increased levels of cell death were observed in FA cell cultures. HU induces DNA damage and potentiates pre-existent damage in FA cells by preventing the translocation of the RNR component p53R2 into the nucleus and activating checkpoint recovery, thus allowing the survival of a proportion of FA cells that have adapted to DNA damage.

## 1. Introduction

Fanconi anemia (FA) is a rare disease, with an incidence of 1 per 360,000 live births; FA is characterized by congenital malformations, bone marrow failure leading to pancytopenia, and a high risk of developing neoplasms [1]. FA cells are highly sensitive to alkylating agents, such as MMC, which induces covalent crosslinks in DNA (ICLs) and generates a high frequency of CAs [2] and is therefore used in the diagnosis of FA [3]. FA is caused by inherited pathogenic variants in one of the 23 genes involved in the FA/BRCA pathway [4,5], which is essential for the repair of interstrand crosslinks (ICLs) by homologous recombination (HR), a highly reliable and error-free process [6]. To repair ICLs in the absence of a normal FA/BRCA pathway, FA cells activate alternative DNA repair mechanisms, such as microhomology-mediated end joining (MMEJ) or nonhomologous end joining (NHEJ), which allows FA cells to survive [7,8].

Hydroxyurea (HU) is a compound to which FA cells are sensitive [9,10]; it is an inhibitor of ribonucleotide reductase (RNR) [11], the enzyme responsible for deoxyribonucleotide triphosphate (dNTP) synthesis, which are essential nucleotides used during DNA replication and repair [12]. The RNR is a tetramer composed of two major subunits (R1) and two minor subunits (R2 or p53R2). The canonical R2 form is limited to the S phase of the cell cycle, whereas the p53R2 subunit (encoded by the *RRM2B* gene) can be found throughout the cell cycle and translocate from the cytoplasm to the nucleus to assemble with R1, an active RNR that provides dNTPs in situ for DNA repair [13].

HU is used for the treatment of different types of cancer, leukemias and sickle cell anemia, and various genotoxic mechanisms have been described for this compound, including depletion of dNTP pools, conflicts between replication and transcription, R-loop formation, and DNA damage by reactive oxygen species (ROS), among others [14,15,16,17]; all these mechanisms imply replication stress. As an old antitumor drug, the effects of HU on DNA replication and the consequent DNA damage in the S phase are well known [18,19]; however, the effects of HU may not be limited to the S phase. In fact, an increase in the frequency of CAs has been observed when FA cells are traversing the G2 phase of the cell cycle and are treated with HU; this effect is enhanced if the cells were previously treated with MMC. Under these conditions, we have observed that practically 100% of the cells exposed to MMC followed by HU display a reduction in the frequency of radial figures and a dramatic increase in the frequency of chromosome breaks [20]. The mechanisms underlying this potentiation of the MMC-induced DNA damage by the addition of HU in the G2 phase are still unclear; however, these data suggest that HU interferes with DNA repair in G2 in a manner that could be related to the localization of RNR at sites of DNA damage, where dNTP availability is needed along with DNA repair proteins.

It is also known that FA cells carrying a large number of CAs might adapt to DNA damage to survive. This adaptation to DNA damage involves bypassing the G2/M checkpoint through a mechanism known as checkpoint recovery (CHKREC), thereby allowing a significant proportion of DNA-damaged cells to escape apoptosis [21].

The aim of this study was to investigate the effect of HU on the DNA repair capacity of FA cells. Specifically, we wanted to determine whether HU, applied in the G2 phase of the cell cycle, generates new DNA damage and/or interferes with the repair of the DNA damage previously induced by MMC. This will contribute to obtaining a better understanding of the process by which FA cells survive and reach mitosis despite the presence of unrepaired DNA damage. We found that HU itself can induce DSBs, which are observed as ɣH2AX signaling during interphase and chromatid breaks in metaphase. HU prevented translocation of p53R2, a DNA damage response subunit of the RNR complex, into the nucleus of damaged FA cells and decreased ɣH2AX signaling, indicating activation of the CHKREC process. The latter would allow FA cells to adapt to DNA damage and divide despite high levels of chromosome damage.

## 2. Results

### 2.1. Hydroxyurea Generates DSBs During the Post-Replicative Stage

**Figure 1:**
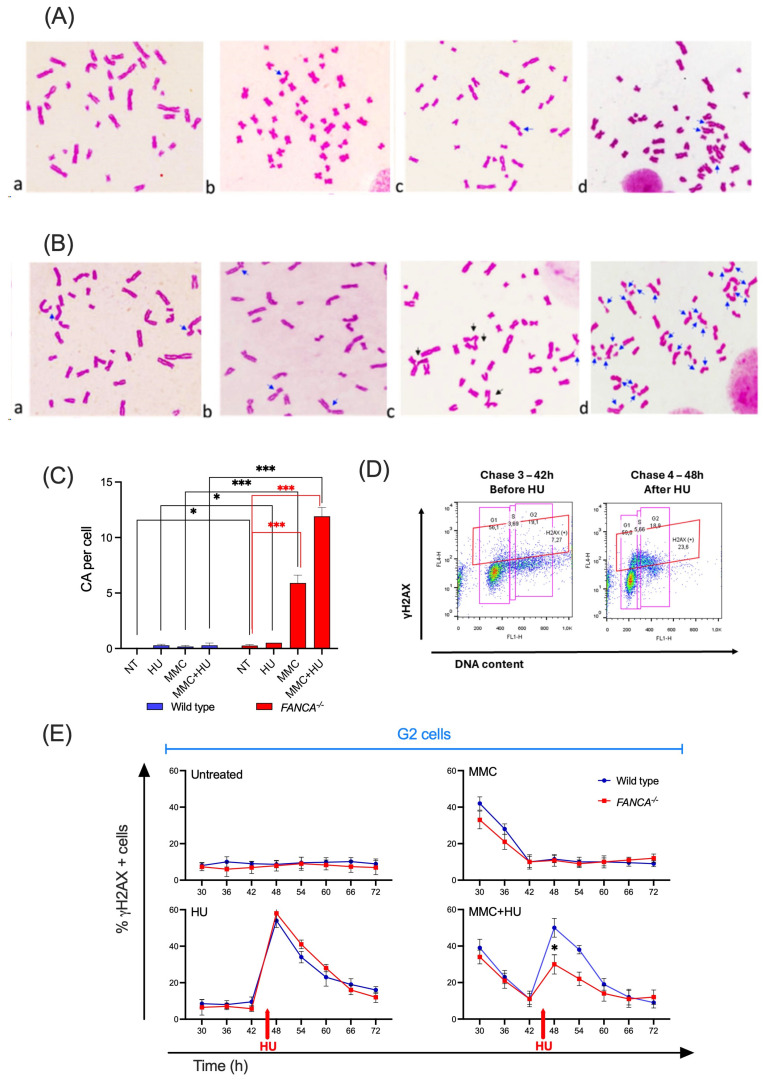
Sequential treatment with MMC followed by HU increases the amount of unrepaired DNA damage in FA lymphoblasts. Cell culture and treatment were performed as described in methodology Section 4.2 and Section 4.3. (**A**) Metaphase spreads showing the presence of CAs in wild-type cells and (**B**) in FA cells. (**a**) Untreated cells (NT); (**b**) cells treated with only HU for 3 h; (**c**) cells treated only with MMC for 24 h from the start of culture; (**d**) sequential treatment with MMC for the first 24 h and then HU the last 3 h. Blue arrows indicate chromatid breaks and black arrows indicate radial figures. All metaphases were observed with a bright field microscope, 100× objective. (**C**) Frequency of chromosomal aberrations per cell. HU, MMC and sequential treatment with MMC + HU significantly increased the amount of CAs in FA cells. Intra-cell line comparisons with statistical significance are represented with red lines and Inter-cell line comparisons with statistical significance are represented with black lines. (**D**) Representative flow cytometry plot showing the percentage of ɣH2AX(+) FA cells before (**left**) and after (**right**) HU treatment. (**E**) Cell cultures were treated as described in methodology Section 4.3 and Section 4.5, and after 30 h of culture, the cells were chased every 6 h; ɣH2AX-positive cells were identified using flow cytometry. Only cells in the G2 phase of the cell cycle that were positive for ɣH2AX are shown. The percentage of ɣH2AX-positive FA cells was significantly lower (*p* = 0.0137) than that in wild-type cells after MMC + HU treatment. All experiments were performed in triplicate. * *p* < 0.05 and *** *p* < 0.001.

The effects of HU during the S phase of the cell cycle are well known and include reduced dNTP supply, replication fork arrest, and replication stress. In this study, we observed that when administered during the *G2* phase of the cell cycle, HU induced chromosomal aberrations, particularly chromatid breaks (Table 1). In wild-type cells not treated with HU, virtually no DSBs were detected (Table 1). In contrast, following HU treatment, FA cells exhibited significantly greater DNA damage than wild-type cells. Regarding 24 h treatment of MMC, wild-type and FA cells responded as expected [22], and the sequential treatment with MMC and HU induced a drastic increase in the frequency of CAs/cell and in the number of cells with DNA damage, which was not observed in wild-type cells. As the frequency of CAs is almost twice the sum of the independently induced damage by MMC or HU, we can consider that HU potentiates the amount of DNA damage (Table 1) (Figure 1A–C).

Using flow cytometry, we followed the dynamics of ɣH2AX signaling, a bona fide surrogate marker for DSBs formation, over time and throughout the cell cycle phases in both wild-type and FA cells after treatment with MMC and/or HU. ɣH2AX-positive cells were detected after both MMC and HU exposure, with the signal becoming evident during the G2 phase and returning to basal levels after 18 h of treatment withdrawal (MMC: chase 3, 42 h of culture; HU: chases 7 and 8, 66 and 72 h of culture, respectively) (Figure 1D,E). Following MMC treatment, ɣH2AX levels initially increased and subsequently declined in both wild-type and FA cells; such a decline in wild-type cells is due to the repair of DNA damage through homologous recombination, whereas in FA cells, this reduction in ɣH2AX signaling might be due to activation of alternative low-fidelity DNA repair pathways or due to inactivation of the ɣH2AX signal. HU treatment rapidly increased the proportion of ɣH2AX-positive cells in the G2 fraction of both cell lines (Figure 1E), indicating that HU induces DSBs during the post-replicative stage (Figure 1E). This is consistent with the notion that, under conditions of mild replication stress, very late replication sites can continue DNA synthesis throughout *G2* until the G2/M transition [23].

After three hours of HU treatment, FA cells previously treated with MMC entered the M phase with markedly increased chromatid breaks (Figure 1B,C). Because chromatid breaks represent unrepaired DSBs, a corresponding increase in ɣH2AX levels would be expected in the interphase G2 FA cells. However, sequential MMC + HU treatment resulted in a low proportion of FA ɣH2AX-positive cells despite the extensive accumulation of chromatid breaks (Figure 1D,E), suggesting that ɣH2AX is actively inactivated in the absence of effective DNA repair.

### 2.2. HU Disrupts Formation of the RNR Complex

HU induces DNA damage during the G2 phase of the cell cycle, as shown by increased chromatid breaks and ɣH2AX activation. Interestingly, although ɣH2AX activation decreases over time, FA cells continue to divide with an extremely high degree of chromosome breakage. This prompted us to hypothesize that HU might interfere with DNA repair through two mechanisms: (1) hindering the recruitment of DNA repair proteins and (2) activating ɣH2AX silencers, allowing the division of cells with DNA damage.

**Figure 2:**
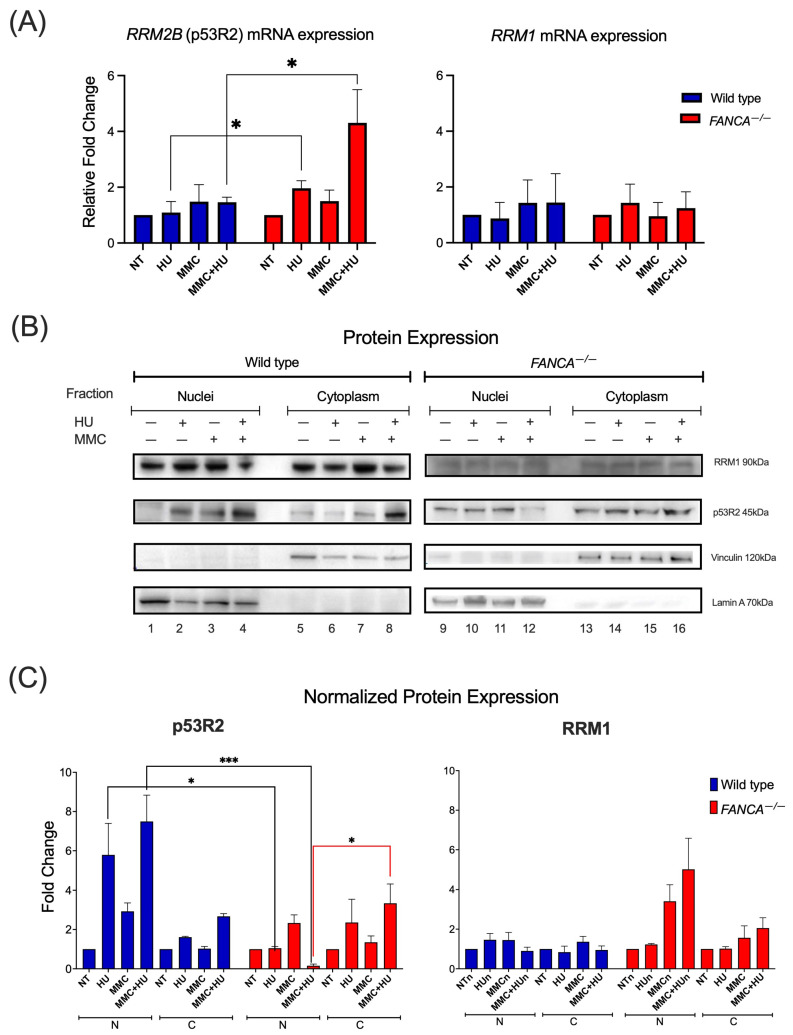
HU affects the expression of ribonucleotide reductase components. (**A**) RT–qPCR expression analysis of ribonucleotide reductase complex components (*RRM2B* and *RRM1*) in wild-type and FA cells exposed to MMC and/or HU. (**B**) Western blot analysis of lysates of nuclear and cytoplasmic fractions of wild-type and FA cells exposed to MMC and/or HU. Data from a representative experiment are shown (n = 2). Densitometry measures were performed using the original membranes. (**C**) Protein quantification of lysates of nuclear and cytoplasmic fractions of wild-type and FA cells exposed to MMC and/or HU. * *p* < 0.05, *** *p* < 0.001.

RNR is an enzyme responsible for synthesizing the dNTPs required for DNA replication and repair. RNR translocates into the nucleus in the presence of DNA damage and localizes to sites of damaged DNA as a prerequisite for effective DSBs repair. As HU targets RNR function, we first assessed its effect on the formation of this complex. First, we assessed whether HU reduced the genetic expression of the components of the RNR complex (*RRM2B* and *RRM1* genes) using RT-PCR. *RRM1* expression did not differ significantly between *FANCA*^−/−^ and wild-type cells under any treatment condition. In contrast, *RRM2B* (p53R2) expression was significantly higher in *FANCA*^−/−^ cells than in wild-type cells following treatment with HU, either alone or in combination with MMC (Figure 2A).

Next, we assessed whether HU affected the translocation of RNR into the nucleus. We treated wild-type and FA cells with MMC and HU, isolated the nuclear and cytoplasmic fractions and assessed the presence of RNR components in both fractions using Western blotting. After DNA damage, we observed a clear translocation of RNR components from the cytoplasm to the nucleus in wild-type cells, especially p53R2, whereas FA cells were deficient in the translocation of RNR components to the nuclear compartment, especially for the p53R2 subunit (Figure 2B,C). These results imply that HU prevents the translocation and assembly of the RNR complex in the nucleus; therefore, the dNTPs required for DNA repair are not available.

### 2.3. WIP1 Phosphatase Is Overexpressed in the Presence of HU

HU interferes with DNA repair by preventing the translocation of the RNR complex from the cytoplasm to the nucleus, thus increasing the number of chromosome breakages, which correlates with the abrupt increase in ɣH2AX signaling upon HU exposure. However, when the cells received MMC-only treatment, ɣH2AX signaling decreased over time, indicating that wild-type cells can repair DSBs and undergo mitosis with minimal chromosome damage. Interestingly, FA cells also showed a reduction in ɣH2AX signaling but displayed a high number of chromatid breaks in metaphase spreads after 48 h of culture, suggesting that the combined treatment interferes with additional signaling mechanisms (Table 1 and Figure 1C).

**Figure 3:**
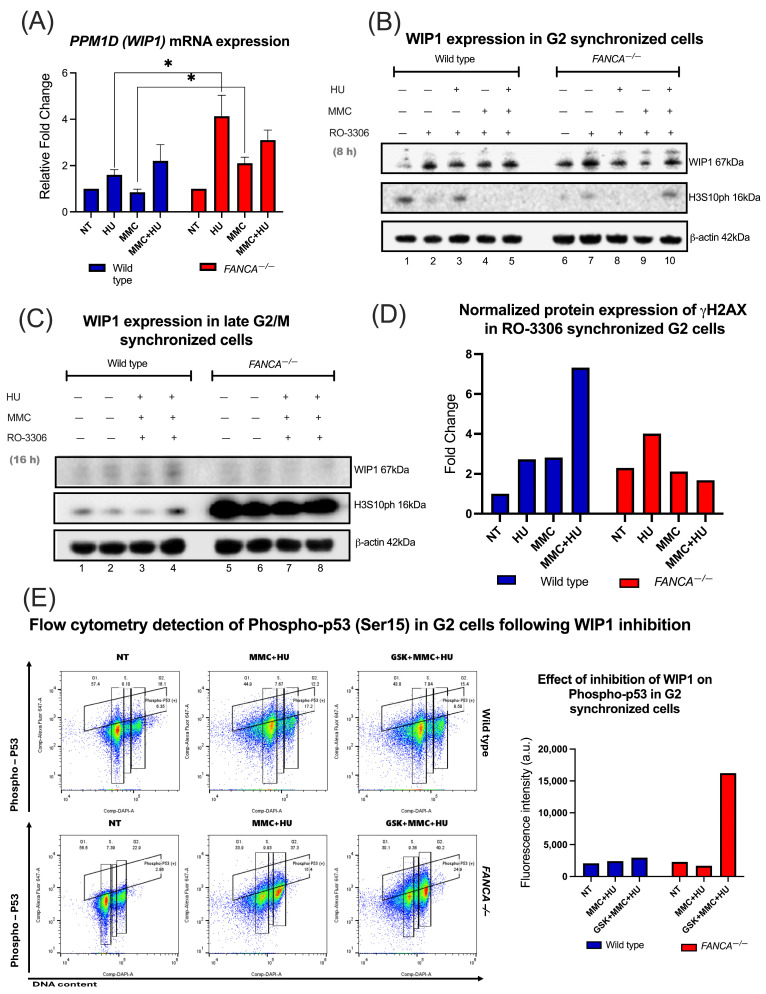
HU induces the expression of the WIP1 phosphatase in FA cells. (**A**) RT–qPCR expression analysis of *PPM1D* (*WIP1*) in wild-type and FA cells exposed to MMC and/or HU. * *p* < 0.05. (**B**) Western blot of lysates of wild-type and FA cells exposed to MMC and/or HU and treated for 8 h with the CDK1 inhibitor RO-3306 (G2 synchronization). (**C**) Western blot of lysates of wild-type and FA cells exposed to MMC and/or HU and treated for 16 h with the CDK1 inhibitor RO-3306 (late G2/M phase synchronization). (**D**) ɣH2AX protein expression; representative Western blot graph of protein lysates (one replicate), from RO-3306 G2-synchronized wild-type and FA cells exposed to MMC and/or HU. (**E**) Representative flow cytometry analysis of phospho-p53 (Ser15) in RO-3306 G2-synchronized cells treated with MMC + HU, with or without the WIP1 inhibitor GSK2830371.

In wild-type cells, the presence of post-replicative DNA damage activates the G2 checkpoint to allow post-replicative DNA repair, and once repaired, the CHKREC process is activated, allowing the cells to progress into mitosis. FA cells are able to activate the CHKREC process even without having performed DNA repair, thus adapting to genomic damage to survive [24]. As we observed that FA cells divide with high levels of chromosome breakage after HU, we tested whether sequential treatment with MMC + HU increased the expression of the WIP1 phosphatase, a well-known CHKREC component that inactivates ɣH2AX through dephosphorylation. We effectively found increased *WIP1* mRNA expression (Figure 3A); however, after synchronizing the cells in G2, using the CDK1 inhibitor RO-3306, we observed similar levels of the WIP1 phosphatase in both wild-type and FA cells. In fact, the amount of ɣH2AX decreases in the G2-synchronized FA cells treated with MMC + HU (Figure 3B,D), which concurred with the results presented in Figure 1E. Likewise, when the G2-synchronized FA cells exposed to MMC + HU were treated with the WIP1 inhibitor GSK2830371, an increase in phospho-p53 (Ser15) was observed (Figure 3E), indicating that FA cells ignore the presence of unrepaired DNA damage and allow entry into mitosis, as denoted by the presence of the M phase-specific H3S10ph histone post-translational modification and the reduction in WIP1 in FA cells mostly enriched in the M phase (Figure 3C).

### 2.4. The G2 Phase Was Extended, and Cell Death Increased After MMC and HU Treatments

**Figure 4:**
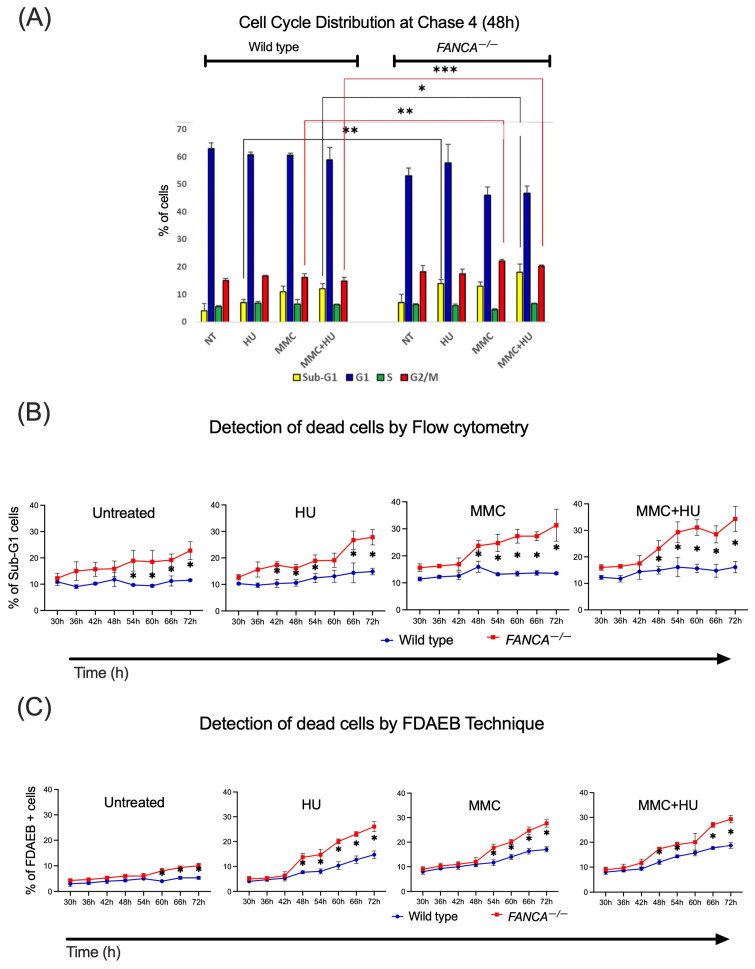
Sequential treatment with MMC followed by HU increased FA cell death. (**A**) Cell cycle distribution of the wild-type and FA cells was analyzed after 48 h of culture. (**B**) Wild-type and FA cells were treated with MMC and/or HU, cell cultures were chased every 6 h, and the sub-G1 phase was analyzed with flow cytometry. (**C**) The percentage of cells positive for the cell death marker FDAEB (fluorescein diacetate and ethidium bromide) was quantified. * *p* < 0.05; ** *p* < 0.01; *** *p* < 0.001.

Although FA cells were able to escape the G2/M checkpoint after exposure to HU, these cells are known for activating subsequent p53-dependent pathways that ultimately kill cells carrying excessive DNA damage. We confirmed increased cell death after MMC and HU treatment by assessing DNA content using flow cytometry, where we observed the cell cycle distribution of FA and wild-type cells after 48 h of culture (Chase 4). In FA cells treated with MMC and MMC+ HU, an increase in the number of cells in the G2 phase (*p* = 0.0078) confirmed activation of the G2/M checkpoint (Figure 4A).

In FA cells, an increase in the sub-G1 fraction after multiple treatments confirmed cell death (Figure 4B), whereas in wild-type cells, the increase in sub-G_1_ was minimal. Using an additional FDAEB-based fluorescence assay, we confirmed this increase in cell death in FA cells over time (Figure 4C). Again, the highest number of dead cells was found in the FA cells treated with MMC + HU, confirming the flow cytometry findings.

## 3. Discussion

In the presence of HU, proliferating cells are arrested in the S phase due to the reduced dNTP availability, which slows DNA polymerase progression at replication forks. In contrast, the effects of HU during post-replicative phases remain largely unexplored. Although numerous studies have reported that HU induces DNA damage in the S phase, its ability to induce DSBs formation during G2/M has remained largely unexplored. Here, we found that HU administered in the G2 phase induces DSBs, as evidenced by the presence of chromosome breaks and ɣH2AX activation in both wild-type and FA cells. Moreover, our results show that during G2 HU interferes with the repair of MMC-induced DNA damage in FA cells. We also observed a marked increase in both cell death and evidence consistent with checkpoint recovery in FA cells exposed to sequential MMC-HU treatment. Together, these findings support a model in which impaired DNA repair and checkpoint recovery promote the persistence of DNA damage and the survival of a subset of FA cells, helping to explain the marked increase in MMC-induced chromosomal damage observed after sequential HU treatment.

### 3.1. HU Treatment in the G2 Phase Induces DSBs and Potentiates Pre-Existing DNA Damage in FA Cells

Consistent with previous reports, we observed a low but reproducible increase in CAs following HU treatment during G2 in both wild-type and FA cells (Table 1) [20]. Although HU is classically considered an S phase-specific inhibitor of DNA synthesis, our results indicate that it also induces DSBs during G2, as evidenced by increased chromatid breaks and ɣH2AX-positive cells. This effect is likely related to delayed completion of DNA replication at common fragile sites (CFSs), which extends into G2 and may lead to replication fork collapse and DSBs formation [25]. In wild-type cells, DSBs decrease over time and are resolved mainly through homologous recombination (HR) [26,27]. Accordingly, although HU induces DNA damage during G2, efficient HR-mediated repair in wild-type cells limits the accumulation of DSBs, consistent with the modest increase in chromosome breaks observed in our study.

In FA cells, HU induces higher levels of CAs as compared with wild-type cells. Deficiency of the FA/BRCA pathway compromises both DNA repair and replication fork protection, leading to the accumulation of spontaneous and replication stress-induced chromatid breaks [28,29]. Notably, although HU approximately doubled the frequency of DNA-damaged cells and CAs in FA cells compared with wild-type cells, the proportion of ɣH2AX-positive cells remained similar, suggesting impaired DNA damage signaling. Together, these findings suggest that ɣH2AX signaling is differentially regulated relative to chromosomal damage accumulation in FA cells.

### 3.2. HU Interferes with the DNA Damage Repair in MMC-Pretreated Cells

We observed that the sequential treatment of MMC and HU potentiated DNA damage in FA cells. FA cells are unable to efficiently repair MMC-induced ICLs; however, they can process the first steps of ICLs repair through alternative DNA nucleases, such as FAN1 [30], which unhook the ICLs and generate DSBs. These breaks are subsequently rejoined through error-prone pathways such as microhomology-mediated end joining (MMEJ) [31]. Little homology is required for this repair pathway; therefore, some of the misjoined DSBs are converted into gross CAs, such as radial figures that are typically observed in MMC-treated FA cells [22,32]. After double MMC + HU treatment, we observed that the increase in CAs was primarily due to chromatid breaks, rather than radial figures, indicating that a greater proportion of DSBs remained unrepaired (Figure 1A–C). One possible explanation is that DSBs rejoining requires a local supply of dNTPs generated by RNR, whose activity is inhibited by HU [33].

Activated p53 (phospho-p53 Ser15) coordinates the cellular response to DNA damage [34] and induces the expression of *RRM1* and *RRM2B* genes, which encode RNR subunits RRM1 and p53R2, respectively. We found that the expression of these genes was not reduced by either HU or MMC. Rather, HU induced *RRM2B* transcription, resulting in increased *p53R2* expression. Surprisingly, despite this transcriptional induction, p53R2 failed to accumulate in the nucleus of *FANCA*^−/−^ cells (Figure 2A–C), where it associates with the RRM1 subunit to form the active RNR complex [35], required to provide dNTPs for efficient DSBs repair through HR or MMEJ [7].

The absence of an active nuclear RNR complex may limit the local availability of dNTPs required for DSBs end rejoining, thereby impairing the repair of MMC-induced lesions. Consistent with this interpretation, we did not observe an increase in radial figures, which represent aberrant repair events involving the rejoining of damaged DNA ends, but rather an accumulation of unrepaired DSBs that become evident as chromatid breaks in mitosis and ɣH2AX signaling during interphase. Thus, impaired RNR activation may contribute to the persistence of MMC-induced DNA lesions and represents an important mechanism by which HU potentiates MMC-induced damage in FA cells. This model also explains why HU exposure primarily increases chromatid breaks rather than radial figures, reflecting a failure to complete DSB repair rather than an increase in erroneous end-joining events.

### 3.3. Heavily DNA-Damaged FA Cells Do Not Display the Expected Signaling by ɣH2AX

We observed a discrepancy between the genomic damage quantified by CAs analysis and DNA damage signaling detected by ɣH2AX, indicating that ɣH2AX levels do not proportionally reflect the extent of chromosomal damage in FA cells. Because ɣH2AX dephosphorylation is associated with DSBs repair [24], the reduced ɣH2AX signal despite extensive chromosomal damage raised the possibility that ɣH2AX signaling is attenuated in surviving FA cells. This observation led us to consider the potential involvement of the CHKREC process in this response. This adaptive response could allow a subset of cells to progress through mitosis carrying unrepaired lesions, thereby promoting genomic instability [32].

### 3.4. FA Cells Activate the Checkpoint Recovery Process to Escape DNA Damage-Induced Cell Death

In wild-type cells, ICLs encountered during the S phase activate the G2/M checkpoint through Ataxia telangiectasia mutated (ATM), Ataxia telangiectasia and Rad3-related (ATR), and p53 and H2A variant X (H2AX) signaling, leading to cell cycle arrest and allowing repair by the FA/BRCA pathway. Such an arrest provides time for ICLs repair by the FA/BRCA pathway, which coordinates ICLs unhooking, generating DSBs, DNA adducts and stretches of single-strand DNA, which will be repaired by HR, nucleotide excision repair (NER) and trans-lesion synthesis (TLS), respectively [36]. Once DNA repair is completed, CHKREC proteins deactivate the G2/M checkpoint, allowing cells to enter mitosis and preventing the transmission of damaged DNA to daughter cells. WIP1 phosphatase is a key CHKREC component that dephosphorylates p53 and other checkpoint proteins [37].

The G2 arrest observed in FA cells (Figure 4A) is due to the action of the transcription factor p53 activated by DNA damage [37]. Under conditions of low DNA damage, p53 acts by inducing a checkpoint that arrests the cell cycle to elicit DNA repair. In the scenario of a high amount of DNA damage, p53 activates the apoptotic pathway [32]. In this study, we have shown that in response to sequential MMC + HU treatment, FA cells arrest the cell cycle in the G2 phase, but this seems not to be enough to repair the excessive amount of DSBs. Under these conditions, extensive cell death would be expected, but this is not occurring.

After 16 h of treatment with RO-3306, and in comparison to wild-type cells, FA cells display an exacerbated accumulation in the mitotic antephase, as indicated by the presence of large amounts of H3S10ph. These findings led us to hypothesize that the CHKREC is forcing FA cells to enter mitosis and accumulate in the M phase. The new arrest at this stage is likely occurring because FA cells are now replicating CFSs, an activity that is very prolonged in FA cells in comparison to wild-type cells [23], and that might prevent mitotic progression (Figure 3C).

Although a significant fraction of FA cells undergoes cell death (Figure 4B,C), many survive. Our observations are consistent with activation of the CHKREC pathway (Figure 3), which promotes dephosphorylation of ɣH2AX and also of p53 through WIP1 [37], as supported by the significant increase in phospho-p53 (Ser15) levels following WIP1 inhibition in G2-arrested FA cells. Attenuation of p53 signaling would be expected to impair p53R2 nuclear translocation and, together with CHKREC-mediated dephosphorylation of ɣH2AX, FA cells may progress through the cell cycle while carrying substantial genomic lesions. This is consistent with previous proposals that CHKREC promotes the survival of heavily DNA-damaged FA cells [24].

Collectively, our findings identify impaired p53R2-mediated RNR regulation and CHKREC activation as key contributors to the accumulation of DNA damage following sequential MMC-HU treatment in FA cells and provide a mechanistic explanation for the previously reported sensitivity to HU in six FA cell lines representing five distinct FA complementation groups [20], thereby contributing to our understanding of the role of defective DNA damage responses in cancer predisposition in FA [32].

We acknowledge the limitations of our study. Our data support a mechanistic model based on the observed experimental evidence rather than demonstrating direct causality. Another limitation of the present study is the use of a single FA cell line.

Future studies should validate these findings in additional FA models and patient-derived samples and incorporate functional experiments to establish the causal roles of p53R2-mediated RNR regulation and CHKREC activation in DNA damage persistence.

## 4. Materials and Methods

### 4.1. Cell Lines

The VU817 cell line is an Epstein–Barr virus (EBV)-immortalized lymphoblastoid cell line established from a female patient (46,XX) with Fanconi anemia complementation group A (FA-A). The cell line carries a pathogenic gross deletion encompassing exon 2 of *FANCA* (NM_000135.4) c.(18_214)_(215_281)del. It was originally established at the Department of Human Genetics, Vrije Universiteit Medical Center (Amsterdam, The Netherlands), by the laboratory of Prof. Hans Joenje and was obtained through scientific collaboration. To the best of our knowledge, VU817 has not been deposited in a public cell repository and therefore has no public accession number or Research Resource Identifier (RRID).

The NL53 cell line is an Epstein–Barr virus (EBV)-immortalized lymphoblastoid cell line established in our laboratory from an anonymized female donor using residual peripheral blood samples obtained from the Blood Bank of the Instituto Nacional de Pediatría (Mexico City, Mexico). The donor had no known hematological or genetic disorder and a normal female karyotype (46,XX). The cell line was generated for research purposes and is maintained in our laboratory. It has not been deposited in a public cell repository and therefore has no public accession number or RRID.

### 4.2. Cell Cultures

The wild-type lymphoblastoid cell line NL-53 (WT) (developed in our laboratory) and the Fanconi anemia lymphoblastoid cell line VU-817, FA-A complementation group (*FANC^−/−^*) (kindly donated by Dr. Hans Joenje, VU University Medical Center, Amsterdam, The Netherlands), were used. Both cell lines were cultured in an incubator with 5% CO_2_ at 37 °C, with a cell density of 300,000 cells/mL, in RPMI 1640 medium (Gibco, BRL, Grand Island, NY, USA) supplemented with 10% inactivated fetal bovine serum (HyClone, Logan, UT, USA) and glutamine, sodium pyruvate, nonessential amino acids, and 1% antifungal–antibiotic solution (Gibco, BRL, Grand Island, NY, USA).

### 4.3. Treatments and Cell Synchronization

**Figure 5:**
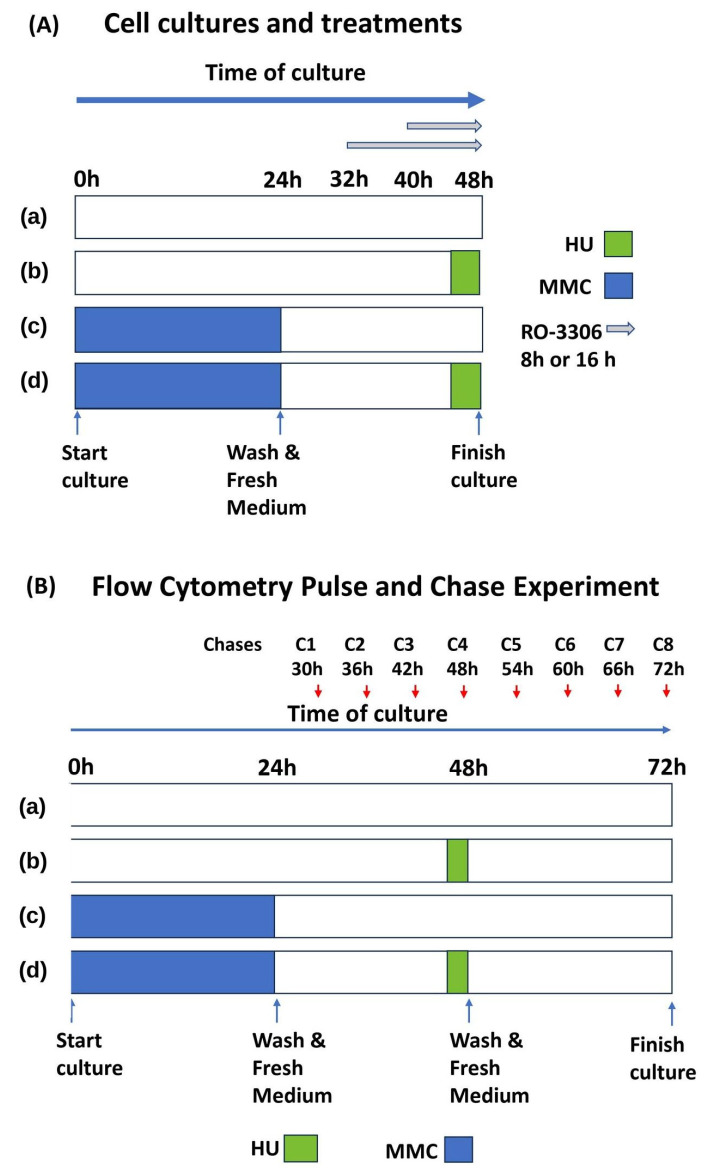
Treatment strategy. (**A**) Cell culture conditions and treatments: (**a**) untreated cells, (**b**) cells treated with HU [2 mM] for 3 h and after 45 h of culture, (**c**) cells treated with MMC [10 ng/mL] for 24 h from the start of culture, and (**d**) cells treated with MMC + HU. Blue arrow indicates the cell culture total time. Gray arrows indicate CDK1 inhibitor RO-3306 treatment time. (**B**) Pulse-chase experiment to analyze the signaling of DSBs by ɣH2AX in the G2/M phase of the cell cycle using flow cytometry; every 6 h (red arrows), cells were chased starting at 30 h and ending at 72 h of culture.

For each cell line, four types of culture conditions were generated (Figure 5A). To assess the effect of HU during the G2 post-replicative phase of the cell cycle, lymphoblastoid cell cultures were exposed to HU [2 mM] (Sigma, St. Louis, MO, USA) for 3 h and then processed according to the requirements of each analysis. The HU working concentration (2 mM) was selected based on a preliminary dose–response experiment in the wild-type NL-53 and Fanconi VU-817 cell lines (Supplementary Figure S1).

To study the effects of HU on FA cells with previously damaged DNA, cells were treated with MMC [10 ng/mL] for 24 h, washed twice with Hank’s balanced solution and then allowed to recover for 24 h in normal fresh medium (Figure 5A(c,d)). HU [2 mM] was added for the final 3 h of culture (Figure 5A(b)), and cell cultures were harvested with colchicine to obtain metaphase chromosomes. This strategy allowed us to obtain metaphase spreads from cells that were exposed to MMC during the S phase and to HU during the G2 phase. This treatment scheme ensures that the analyzed metaphase spreads belong to cells that were traversing the G2 phase of the cell cycle during the 3 h of exposure to HU.

Additionally, to evaluate the expression of *WIP1* during the G2 phase of the cell cycle, both cell lines were synchronized in G2 using the CDK1 inhibitor RO-3306 [8 μM] (Merck-Millipore, Darmstadt, Germany) for 8 h or for 16 h to obtain cells in the early M phase (Figure 5A, grey arrows). The state of ɣH2AX in G2- synchronized cells was assessed using protein expression analysis. In addition, to show that *WIP1* is inactivating the checkpoint, cells were treated with GSK2830371 [14 µM], a WIP1 inhibitor, and the level of phospho-p53 (Ser15) was assessed by flow cytometry in the cells with double treatment MMC + HU.

### 4.4. Chromosomal Aberrations

For chromosomal aberration analysis, colchicine (Sigma–Aldrich, St. Louis, MO, USA) was added at a concentration of 1 μg/mL to cell cultures 2 h before collection. Cells were treated with 0.075 M KCl (Sigma, St. Louis, MO, USA) and fixed in 3:1 methanol and glacial acetic acid (Sigma, St. Louis, MO, USA). The fixed cells were placed on slides and stained with Giemsa (Merck, Darmstadt, Germany) and Wright (Merck, Darmstadt, Germany) in Sörensen buffer. The number of chromatid breaks, chromosome breaks and radial figures were scored at 50 metaphases per treatment. Each experiment was replicated three times.

### 4.5. Flow Cytometry and Pulse-Chase Experiments

Pulse-chase experiments were carried out according to Figure 5B. Eight pulse-chase time points were analyzed from 30 to 72 h of culture every 6 h. To analyze the cell distribution in the different phases of the cycle, the DNA was stained with Sytox Green (Thermo Fisher, Waltham, MA, USA) and analyzed by flow cytometry. To determine the proportion of cells in the G2 phase of the cell cycle that were positive for ɣH2AX as an indicator of DSBs, an anti-ɣH2AX antibody conjugated with Alexa 648 fluorochrome (Becton Dickinson, Ashland, OR, USA) was used. Flow cytometry procedures were performed with a FACSCalibur cytometer (Becton Dickinson, Ashland, OR, USA). Cells were fixed with Cytofix/Cytoperm solution (Becton Dickinson, Ashland, OR, USA) and then acquired in PBS at a cell density of 5 × 10^5^–1 × 10^6^ cells/mL. Fluorescence signals for cell cycle distribution, sub-G1 population and ɣH2AX positivity in G2/M cells were outlined in specific gates and quantified using the FlowJo software (v10.8.1). Cells located in the sub-G1 region of the cell cycle were considered dead cells. Post-replicative DNA damage was assessed only in cells that, due to their DNA content, were traversing the G2/M phase. Experiments were performed in triplicate. A second set of flow cytometry studies was performed to show the checkpoint inhibition by WIP1. WIP1 inhibitor GSK2830371 [14 µM] (Sigma–Aldrich, St. Louis, MO, USA) was used, and the levels of phospho-p53 (Ser15) were studied in cells synchronized in the G2 phase with RO-3306 for 8 h, the DNA was stained with DAPI (Becton Dickinson, Ashland, OR, USA), and the status of phospho-p53 (Ser15) was determined only in the G2/M cells. Phospho-p53 (Ser 15) was detected with an anti-phospho-p53 (Ser15) mAb (Cell Signaling, Danvers, MA, USA) and Alexa 488 secondary antibody (Invitrogen, Waltham, MA, USA). These experiments were acquired in a Cytek Northern Lights full-spectrum flow cytometer (Cytek Biosciences, Fremont, CA, USA). Positivity in G2/M cells was outlined in specific gates and quantified using FlowJo software (v10.10.0).

### 4.6. Reverse Transcription Quantitative Real-Time PCR (RT–qPCR)

For each treatment, total RNA was obtained employing the combined TRIzol method (Invitrogen, Carlsbad, CA, USA) followed by the RNeasy Mini procedure (Qiagen, Valencia, CA, USA) according to the manufacturer’s instructions. Before retro-transcription and to eliminate DNA contamination, one microgram of total RNA was treated with 0.1 U RNase-free DNase I. RNA was reverse transcribed using the Transcriptor First Strand cDNA Synthesis Kit (Roche Diagnostics, GmbH, Mannheim, Germany) using anchored oligo dT and random hexamer primers. Quantitative polymerase chain reaction (qPCR) was performed on cDNA samples per duplicate for each cell line treatment and biological repeat. In all cases, we used 2 µg of cDNA per reaction in Light Cycler 2.0 Carrousel Roche equipment, and the conditions were as follows: 1 cycle at 95 °C for 10 min, 1 cycle at 95 °C for 10 s, and 1 cycle at 60 °C for 30 s, for a total of 45 cycles. The Universal Probes system (Roche Diagnostics, GmbH, Mannheim, Germany) and the Light Cycler Taq Man Master kit (Roche Diagnostics, GmbH, Mannheim, Germany) were used. *RRM1*, *RRM2B* and *WIP1* gene expression was quantified, and *7SL* and *B2M* (β2 microglobulin) were used as reference genes. Primers for each gene were designed online with ProbeFinder Software (https://primers.neoformit.com/) and manufactured at the Sequencing and Synthesis Unit (IBT, UNAM, México). The primer sequence and the universal probe for each gene are indicated in Table A1.

Relative expression levels for each cell line and for each treatment were calculated using the formula of relative expression = 2^−ΔΔCt^ = 2^−(ΔCtsample−ΔCtcalibrator)^ [38].

For each sample, *7SL* and *B2M* reference gene Ct values were utilized for normalization purposes.

### 4.7. SDS–PAGE and Western Blot

Nuclear and cytoplasmic protein extracts were obtained according to the Nabbi and Riabowol protocol [39], and 60 μg of each protein extract and condition was employed. Only for WIP1 protein expression analysis, whole-cell lysates were obtained with RIPA buffer solution (Sigma–Aldrich, St. Louis, MO, USA) with a respective protease inhibitor cocktail (Sigma–Aldrich, St. Louis, MO, USA). Proteins were separated by electrophoresis on 12% SDS–PAGE gels, transferred to a polyvinylidene fluoride membrane (Bio-Rad, Hercules, CA, USA), and blocked with 5% skim milk solution in PBS supplemented with 0.1% Tween 20. Detection of RRM1 (Abcam, Cambridge, UK) and p53R2 (Abcam, Cambridge, UK) was performed with the corresponding primary antibodies. Vinculin (Genetex, Irvine, TX, USA) was used as the loading control for cytoplasmic extracts, and Lamin A (Genetex, Irvine, TX, USA) was used as the loading control for nuclear extracts. The following secondary antibodies were used: goat polyclonal antibody to rabbit IgG H+L HRP conjugate (Promega, Madison, WI, USA) and goat polyclonal antibody to mouse IgG H+L HRP conjugate (Promega, Madison, WI, USA). ɣH2AX protein levels were determined in total protein extracts from RO-3306 synchronized cells, using an anti-gamma phospho-H2AX antibody (Merck-Millipore, Darmstadt, Germany).

### 4.8. Fluorescein Diacetate and Ethidium Bromide (FDAEB) Cell Death Assay

To verify the levels of cell death for both cell lines under the 4 different treatments, the FDAEB cell viability technique was used, according to the protocols of Gray and Morris [40]. This assay was compared with the results obtained by flow cytometry to assess concordance between both techniques.

### 4.9. Statistical Analyses

All statistical analyses were performed using GraphPad Prism software (v. 8.0.2). Data normality was assessed using the Shapiro–Wilk test for small samples. Comparisons among treatments within the same cell line were performed using two-way analysis of variance (ANOVA). Differences between cell lines were evaluated using Student’s *t*-test. Statistical significance was defined as *p* < 0.05.

## 5. Conclusions

Our findings demonstrate that HU treatment during the G2 phase induces DSBs in both wild-type and FA cells, as evidenced by ɣH2AX activation during interphase and chromatid breaks in mitosis. When administered after MMC, HU markedly increased chromosomal damage in FA cells compared with either treatment alone.

Failure of p53R2 to accumulate in the nucleus of *FANCA*^−/−^ cells identifies a key mechanism underlying the enhancement of MMC-induced DNA damage by HU.

HU increased the frequency of DNA-damaged cells and chromosomal aberrations in MMC-pretreated FA cells compared with wild-type cells, whereas the proportion of ɣH2AX-positive cells remained comparable between both cell types. These findings indicate that ɣH2AX activation and chromosomal damage accumulation are not proportionally correlated in FA cells under these conditions.

Defective nuclear accumulation of p53R2 and evidence of CHKREC activation identify two complementary mechanisms underlying DNA damage persistence and the survival of FA cells following sequential MMC-HU treatment.

Together, these findings provide new mechanistic insights into the DNA damage response of FA cells and advance our understanding of the molecular basis of genomic damage in Fanconi anemia.

## Figures and Tables

**Table 1:** Frequency and type of CAs per cell after treatment with MMC and/or HU.

	**Wild-Type Cells (NL-53)**			
	Frequency of CAs (Average ± SD)	% Breaks (Average ± SD)	% Rejoined radial figures (Average ± SD)	% Cells with damage (Average ± SD)
Untreated	0.027 ± 0.02	100 ± 0	0	6 ± 2
HU	0.28 ± 0.11 ^●^	100 ± 0	0	11.3 ± 1.2
MMC	0.17 ± 0.13	100 ± 0	0	5.3 ± 1.2
MMC + HU	0.28 ± 0.16	95.8 ± 7.2 ^●^	4.2 ± 7.2	11.3 ± 2.3 ^●^
	***FANCA^−/−^*** **Cells (VU-817)**			
	Frequency of CAs (Average ± SD)	% Breaks (Average ± SD)	% Rejoined radial figures (Average ± SD)	% Cells with damage (Average ± SD)
Untreated	0.27 ± 0.06 ^▲^	100 ± 0	0	18.7 ± 6.1 ^▲^
HU	0.52 ± 0.04 ^▲^*	98.6 ± 2.5 ^◇^	1.4 ± 2.5 ^◇^	26 ± 4 ^▲^*
MMC	5.91 ± 0.66 ^▲^*	90.2 ± 3.2 ^▲◇^	9.8 ± 3.2 ^▲^*	86 ± 9.2 ^▲^*
MMC + HU	11.91 ± 0.78 ^▲^*	97.2 ± 0.9 ^◇^	2.8 ± 0.9 ^◇^*	100 ± 0 ^▲^

SD = standard deviation. Intra-cell line comparison, wild type ^●^ *p* < 0.05 untreated vs. HU, untreated vs. MMC, HU vs. MMC, MMC vs. MMC + HU. Intra-cell line comparison, *FANCA^−/−^* ^◇^ *p* < 0.05 untreated vs. HU, HU vs. MMC, MMC vs. MMC + HU. * *p* < 0.01 untreated vs. MMC, untreated vs. MMC + HU, HU vs. MMC, HU vs. MMC + HU, MMC vs. MMC + HU. Inter-cell line comparison wild type vs. *FANCA^−/−^* ^▲^ *p* < 0.05 untreated, HU, MMC, MMC + HU.

## Data Availability

The original contributions presented in this study are included in this article. Further inquiries can be directed to the corresponding authors.

## Supplementary Materials

The following supporting information can be downloaded at: https://www.mdpi.com/article/10.3390/ijms27156782/s1.

## Abbreviations

ATM	Ataxia telangiectasia mutated
ATR	Ataxia telangiectasia and Rad3-related
CAs	Chromosomal aberrations
cDNA	Complementary DNA
CHKREC	Checkpoint Recovery
CFSs	Common fragile sites
DSBs	Double-strand breaks
dNTPs	Deoxyribonucleotide triphosphates
FA	Fanconi anemia
FA/BRCA	Fanconi anemia/BRCA DNA repair pathway
FDAEB	Fluorescein diacetate and ethidium bromide
ɣH2AX	Phosphorylated histone H2AX (phosphorylated at serine 139)
H2AX	Histone H2A variant
HU	Hydroxyurea
HR	Homologous recombination
IBT	Instituto de Biotecnología
ICLs	Interstrand crosslinks
MMC	Mitomycin
MMEJ	Microhomology-mediated end joining
NHEJ	Nonhomologous end joining
qPCR	Quantitative polymerase chain reaction
RNR	Ribonucleotide reductase
ROS	Reactive oxygen species
RT–qPCR	Reverse transcription quantitative polymerase chain reaction
SDS-PAGE	Sodium dodecyl sulfate–polyacrylamide gel electrophoresis
TLS	Trans-lesion synthesis
UNAM	Universidad Nacional Autónoma de México

## Appendix A

**Table A1:** Primers and probes used for RT–qPCR.

Gene	Sequence 5′-3′	Universal Probe *
*RRM1*	F CCACATAATGGCAAACACTCTC	49
	R GCAGAATTCAGGCGATCTTT	
*RRMB (p53R2)*	F TTGGTAGTGGACTTGGGAAATC	3
	R AAAGGGAAATGGTGGGAAAC	
*WIP1*	F CCCATGTTCTACACCACCAGT	25
	R TGGTCCTTAGAATTCACCCTTG	
*7SL*	F GCTGGAGGATCGCTTGAGT	26
	R GACACCCGATCGGCATAG	
*B2M*	F TTCTGGCCTGGAGGCTATC	42
	R TCAGGAAATTTGACTTTCCATTC	

F. Forward primer.; R. reverse primer; * number of universal probes (Roche Diagnostics, GmbH, Mannheim, Germany).
