# Supplementary material for: Mechanisms of Hydroxyurea-Mediated DNA Damage Potentiation in Fanconi Anemia Cells
Source: Int J Mol Sci. 2026 Jul 29;27(15):6782. doi: 10.3390/ijms27156782 (PMC13466969; doi:10.3390/ijms27156782)
Supplement: Supplementary file 1 [file ijms-27-06782-s001.zip › ijms-4405662-supplementary materials.pdf]

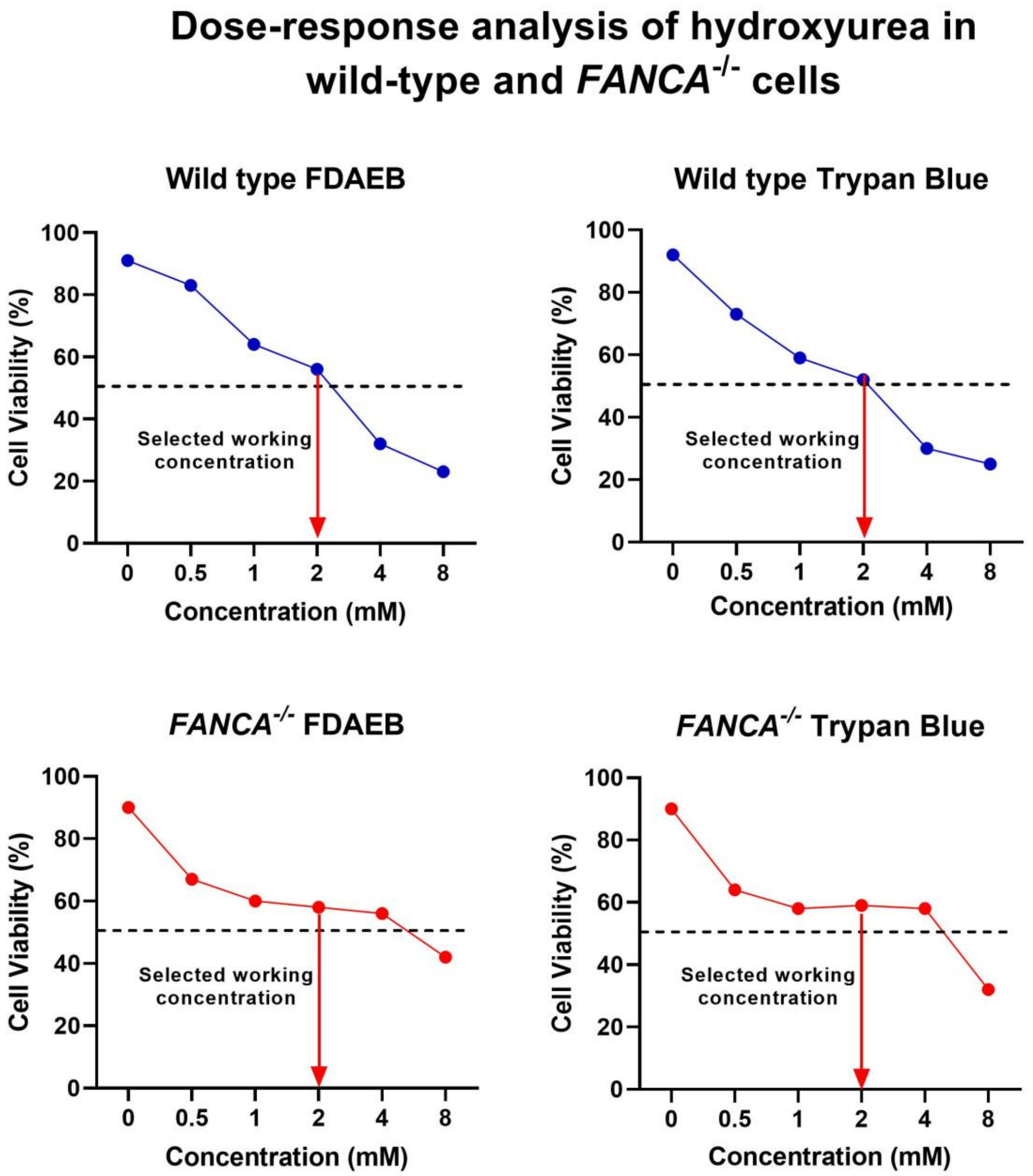

Supplementary Figure S1. **HU dose–response experiment, used to select the hydroxyurea working concentration in the NL-53 and VU-817 cell lines.** Cells were seeded at a density of 3×10^5 cells/mL in 24-well plates and cultured in an incubator at 37 °C with 5% CO_2. Cells were exposed to increasing concentrations of hydroxyurea (0, 0.5, 1, 2, 4, and 8 mM) for 72 h. Cell viability was assessed using both the FDAEB viability assay (see Section 4.7, Materials and Methods) and trypan blue exclusion. Based on the results of this preliminary dose–response experiment, 2 mM hydroxyurea was selected as the working concentration for all subsequent experiments.
